# The biomechanical effect of preexisting different types of disc herniation in cervical hyperextension injury

**DOI:** 10.1186/s13018-021-02677-y

**Published:** 2021-08-24

**Authors:** Jian-jie Wang, Meng-lei Xu, Hui-zi Zeng, Liang-dong Zheng, Shi-jie Zhu, Chen Jin, Zhi-li Zeng, Li-ming Cheng, Rui Zhu

**Affiliations:** 1 Key Laboratory of Spine and Spinal Cord Injury Repair and Regeneration of Ministry of Education, Orthopaedic Department of Tongji Hospital, Tongji University School of Medicine, 389 Xincun Road, 200065 Shanghai, People’s Republic of China; 2 Shanghai Clinical Research Center for Aging and Medicine, Shanghai, 200040 People’s Republic of China

**Keywords:** Cervical hyperextension injury, Spinal cord, Disc herniation, Finite element

## Abstract

**Objective:**

Preexisting severe cervical spinal cord compression is a significant risk factor in cervical hyperextension injury, and the neurological function may deteriorate after a slight force to the forehead. There are few biomechanical studies regarding the influence of pathological factors in hyperextension loading condition. The aim of this study is to analyze the effects of preexisting different types of cervical disc herniation and different degrees of compression on the spinal cord in cervical hyperextension.

**Method:**

A 3D finite element (FE) model of cervical spinal cord was modeled. Local type with median herniation, local type with lateral herniation, diffuse type with median herniation, and diffuse type with lateral herniation were simulated in neutral and extention positions. The compressions which were equivalent to 10%, 20%, 30%, and 40% of the sagittal diameter of the spinal cord were modeled.

**Results:**

The results of normal FE model were consistent with those of previous studies. The maximum von Mises stresses appeared in the pia mater for all 32 loading conditions. The maximum von Mises stresses in extension position were much higher than in neutral position. In most cases, the maximum von Mises stresses in diffuse type were higher than in local type.

**Conclusion:**

Cervical spinal cord with preexisting disc herniation is more likely to be compressed in hyperextension situation than in neutral position. Diffuse type with median herniation may cause more severe compression with higher von Mises stresses concentrated at the anterior horn and the peripheral white matter, resulting in acute central cord syndrome from biomechanical point of view.

## Introduction

Cervical hyperextension injury is a common type of cervical injuries with potentially devastating outcomes [1, 2]. The neck is moving under a rapid, fierce, and backward motion, and it is often accompanied by consequent spinal cord, vertebral, and paravertebral structure injury [3]. The disability caused by cervical hyperextension injury varies from person to person. The symptoms can be mild numbness, or hyperalgesia of the hands, or severe quadriplegia [4]. And the economic impact of this condition is substantial, which is a heavy burden to the patients’ families and the society [5–7].

Since direct in vivo or in vitro measurements of stress and strain are restrictive, a finite element model of spinal cord is helpful to analyze the stress and strain in the cord during compression. The previous study using finite element analysis to study stress distribution in the spinal cord following cervical hyperextension indicated high localized stress at the anterior and posterior horn in the gray matter and the lower cervical levels experienced higher extension motion on acceleration/deceleration of the neck [8–10]. Scifert et al. [11] developed a 3D finite element model of C5-C6 segment to analytically quantify the mechanical response of the spinal cord to cervical spine injury under flexion or extension moments. Greaves et al. [12] compared the cord strain distributions for three injury mechanisms (transverse contusion, distraction and dislocation) by finite element analysis. However, these studies did not consider the potential effect of degenerative changes. Preexisting severe cervical spinal cord compression is a significant risk factor in hyperextension injury and the neurological function may deteriorate after a slight force to the forehead [13–15]. The compressions caused by degenerative factors include herniated cervical intervertebral disc, osteophyte, and incrassate ligaments. And previous studies had shown that patients developed severe paralysis more frequently when the degree of compression was severe above some threshold level [13, 16]. Nishida et al. [17, 18] analyzed the stress distributions of the spinal cord with neck extension with a rigid flat plate by finite element analysis. Nevertheless, there are few biomechanical studies regarding the influence of pathological factors in hyperextension loading condition.

Cervical disc herniation is one of the most common degenerative pathologies of myelopathy or radiculopathy [19]. It can be classified into different types according to the imaging features in the sagittal and axial planes of MRI or CT, local type and diffuse type in the sagittal plane, and median, paramedian, and lateral type in the axial plane, respectively [20, 21]. Different types and different location can cause different syndromes. For example, the median and paramedian herniations can cause the syndromes of spinal cord pressure and the devastating outcomes immediately after the cervical hyperextension injury. And lateral herniation of the cervical spine can cause radiculopathy. However, quantitative analysis for different types and different degrees of compression is rare.

Therefore, the aim of this study is to analyze the effects of preexisting different types of cervical disc herniation and different degrees of compression on the spinal cord in cervical hyperextension loading condition by finite element analysis.

## Material and method

A 3D FE model of the human cervical spinal cord with disc herniation was constructed. MRI images of a 25-year-old male without spine diseases provide the anatomical contour for gray matter and white matter of the spinal cord. The geometry of spinal cord was assumed to be symmetrical about the mid-sagittal plane. The MRI images can not show pia mater clearly [22]; thus, a thin shell with 0.1 mm thichness [23] was set around white matter. A lordotic curevature was obtained from MRI images of the same vollenteer. The 3D model was constructed by extrude the cross section along with the curevature. The model was meshed by 3D solid elements.

The mechanical properties of different components were from previous literatures and listed in Table 1. Ogden’s nonlinear, hyperelastic constitutive model was utilized for the white matter (*μ* = 0.004 MPa, *α* = 12.5) and gray matter (*μ* = 0.0041 MPa, *α* = 14.7) [24, 25] to represent their nonlinear characteristic. Elastic properties (*E* = 2.3 MPa, *υ* = 0.3) was utilized for the pia mater refer to previous literatures [26, 27].

**Table 1:** Mechanical properties of different components used in the present study

Description	Material properties	Density	References
Gray matter	Hyperelastic (Ogden): *μ* = 0.0041 MPa, *α* = 14.7	1050 kg/m^3^	Khuyagbaatar et al. [24]; Ogden et al. [25]
White matter	Hyperelastic (Ogden): *μ* = 0.004 MPa, *α* = 12.5	1050 kg/m^3^	Khuyagbaatar et al. [24]; Ogden et al. [25]
Pia mater	*E* = 2.3 MPa, *υ* = 0.3		Ozawa et al. [26]; Czyz et al. [27]
Vertebral lamina	Rigid body		

Compressive forces of 0–0.08 N were applied in the middle of the spinal cord in a saggital direction on vertical plane. The relationship between the calculated displacement and the compressive force was compared to the data from previous experimental study [28] for validation.

**Fig. 1:**
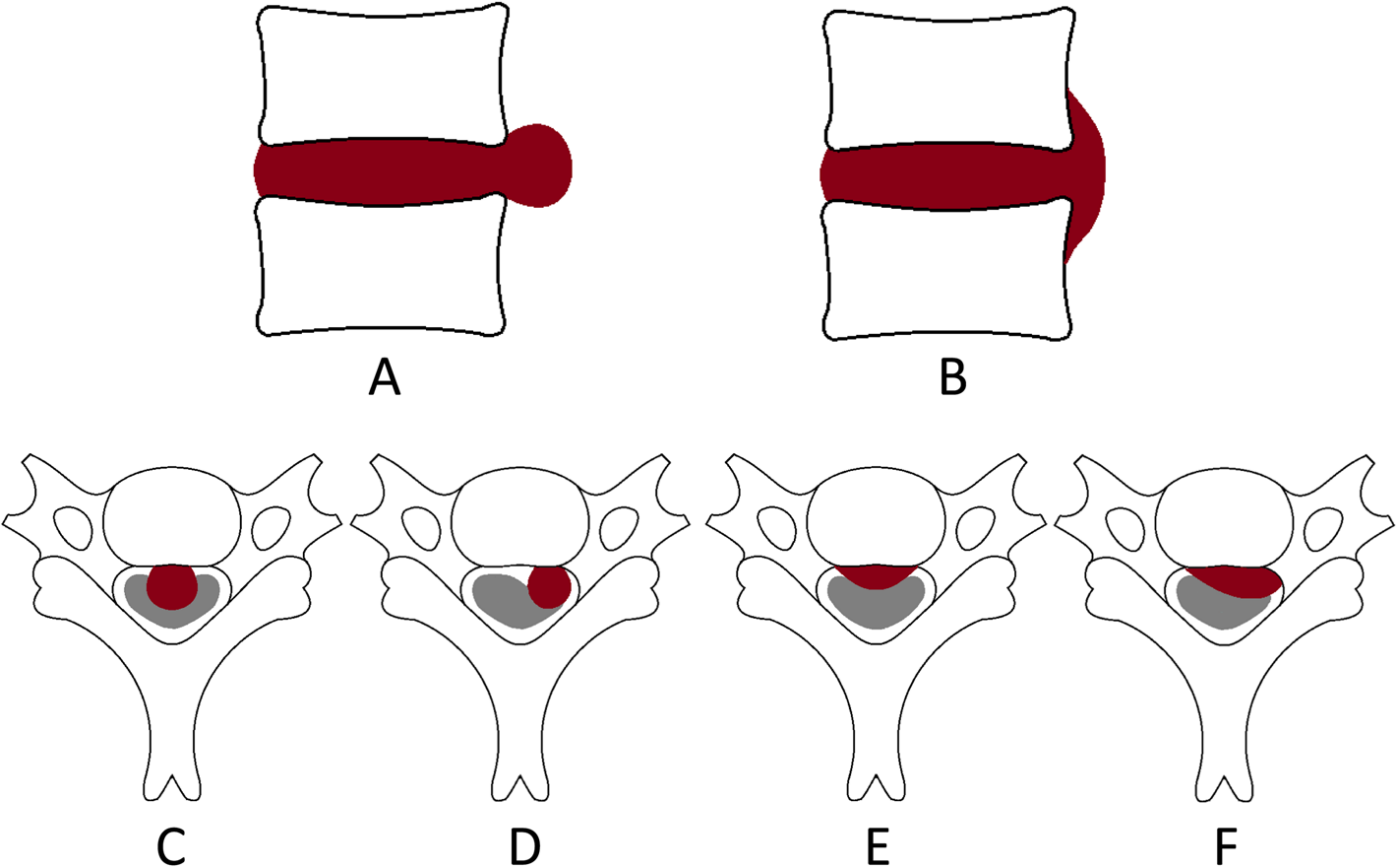
The schematic diagram for different types of disc herniation. **A** Local type. **B** Diffuse type. **C** Local type with median herniation. **D** Local type with lateral herniation. **E** Diffuse type with median herniation. **F** Diffuse type with lateral herniation

**Fig. 2:**
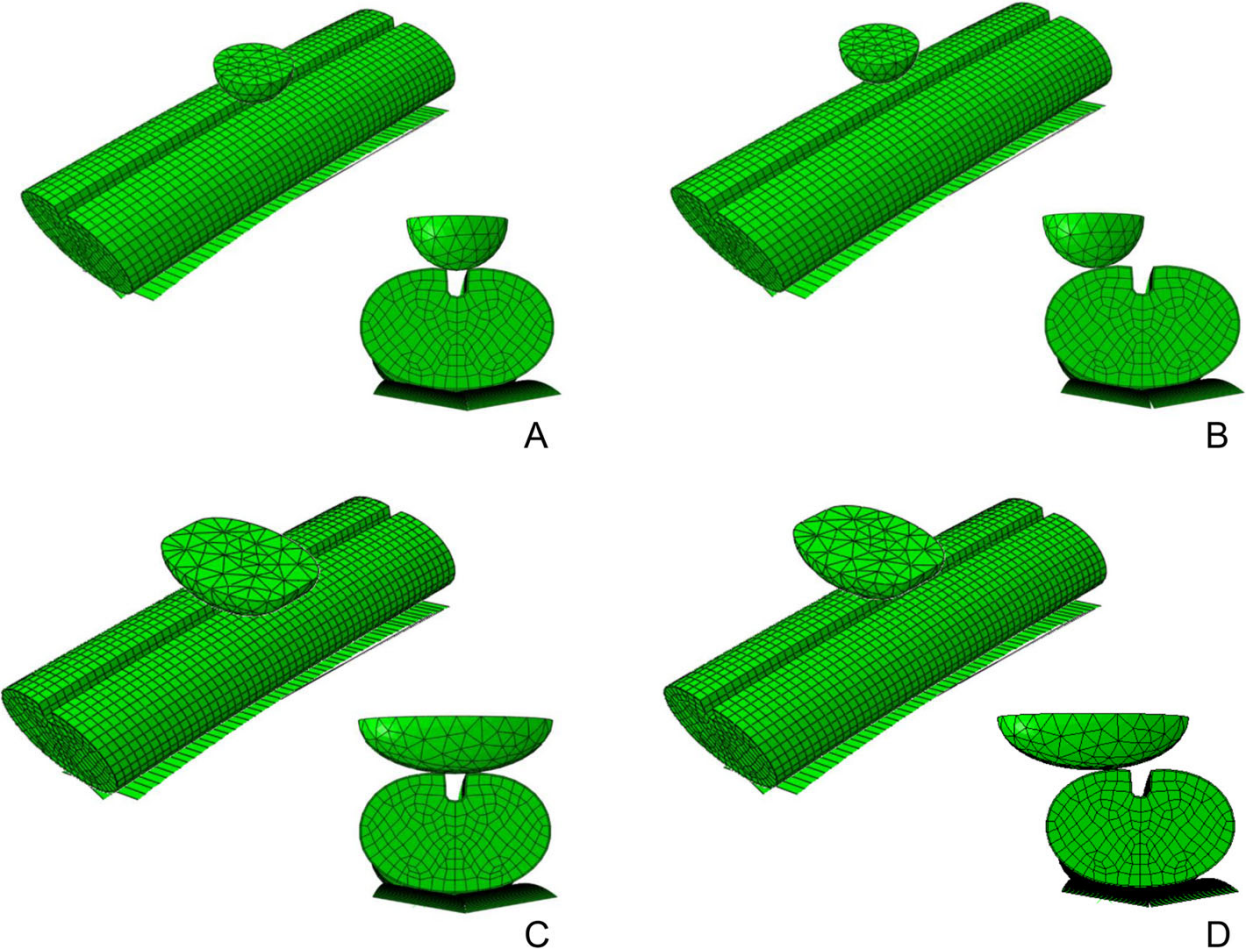
The models simulating different types of disc herniation. **A** Local type with median herniation. **B** Local type with lateral herniation. **C** Diffuse type with median herniation. **D** Diffuse type with lateral herniation

Two types of cervical disc herniation were modeled. The local type was modeled as a hemisphere with the radius of 3.36 mm which equal to 40% sagittal diameter of the spinal cord. The diffuse type has larger contact area and modeled like a pie. The middle cross section area is model as half ellipse with long axis of 13 mm and thickness of 6.72 mm. The valued of long axis is scaled to 100% of the lateral cord diameter and the thickness equals to 40% sagittal diameter of the spinal cord. Local type with median herniation (LTMH), local type with lateral herniation (LTLH), diffuse type with median herniation (DTMH), and diffuse type with lateral herniation (DTLH) were simulated. The schematic diagrams for middle cross section areas and the models were shown in Fig. 1 and Fig. 2.

The ball and pie which represent disc herniation were perpendicular to the cervical spinal cord. A set of displacements, equivalent to 10%, 20%, 30%, and 40% of the sagittal diameter of the spinal cord representing different compression severity, were applied to the disc herniation model to the spinal cord. Besides different types of disc herniation, neutral position and hyperextension position were simulated. Hyperextension position was simulated by backward extending the spinal cord to 20.7° to assume maximum range of motion. This magnitude is adapted by the average value of cervical range of motions from literatures [29–33]. Loading conditions were simulated by combination of four different types of disc herniation and two positions and four compression ratios. The maximum von-Mises stress and stress distributions in different components of the spinal cord were calculated. The FE analyses were carried out in ABAQUS (Valley Street, Providence, RI, USA).

## Results

With compressive forces of 0–0.1 N in the saggital plane, the curve of load versus deformation had a similar trend as the data measured from the in vitro experiment [28]. Detailed data can be found in previous study .

**Fig. 3:**
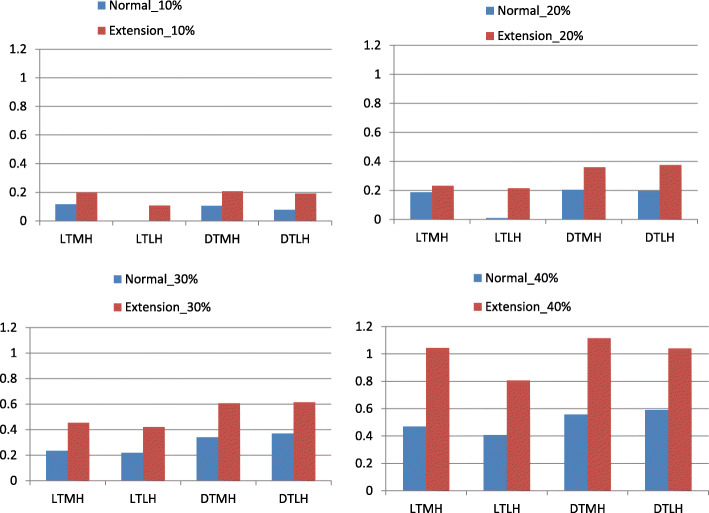
The maximum von Mises stress in the whole model for all loading conditions. LTMH, local type with median herniation; LTLH, local type with lateral herniation; DTMH, diffuse type with median herniation; DTLH, diffuse type with lateral herniation. Unit of stress: MPa

The maximum von Mises stresses in the whole model for all 32 loading conditions were displayed in Fig. 3. The maximum von Mises stresses appeared in the pia mater for all cases. The maximum von Mises stresses in extension position were much higher than in neutral position. In most cases, the maximum von Mises stresses in diffuse type were higher than in local type.

**Fig. 4:**
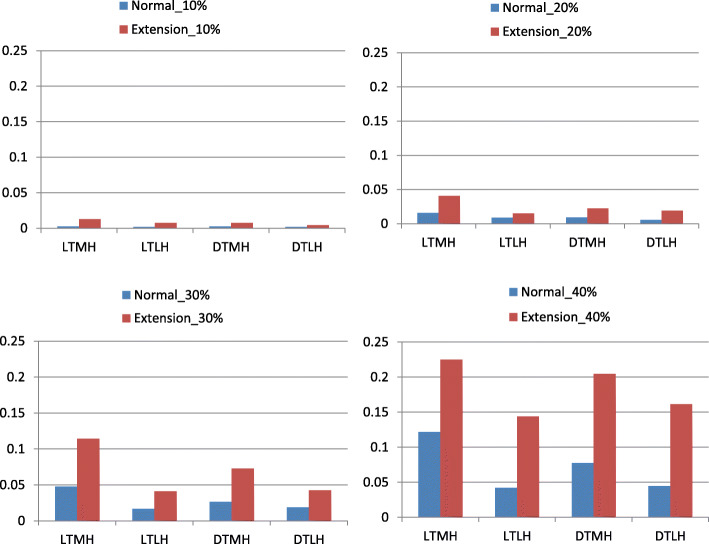
The maximum von Mises stress in the white matter for all loading conditions. LTMH, local type with median herniation; LTLH, local type with lateral herniation; DTMH, diffuse type with median herniation; DTLH, diffuse type with lateral herniation. Unit of stress: MPa

**Fig. 5:**
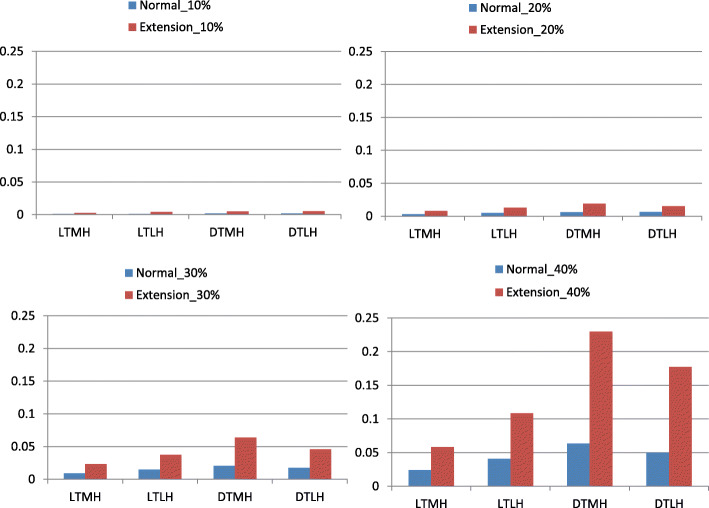
The maximum von Mises stress in the gray matter for all loading conditions. LTMH, local type with median herniation; LTLH, local type with lateral herniation; DTMH, diffuse type with median herniation; DTLH, diffuse type with lateral herniation. Unit of stress: MPa

The maximum von Mises stresses in the white matter and gray matter for all 32 loading conditions were displayed in Fig. 4. and Fig. 5. In any time, the maximum von Mises stresses in extension position were much higher than in neutral position. Local type mainly affected white matter while diffuse type mainly affect gray matter.

**Fig. 6:**
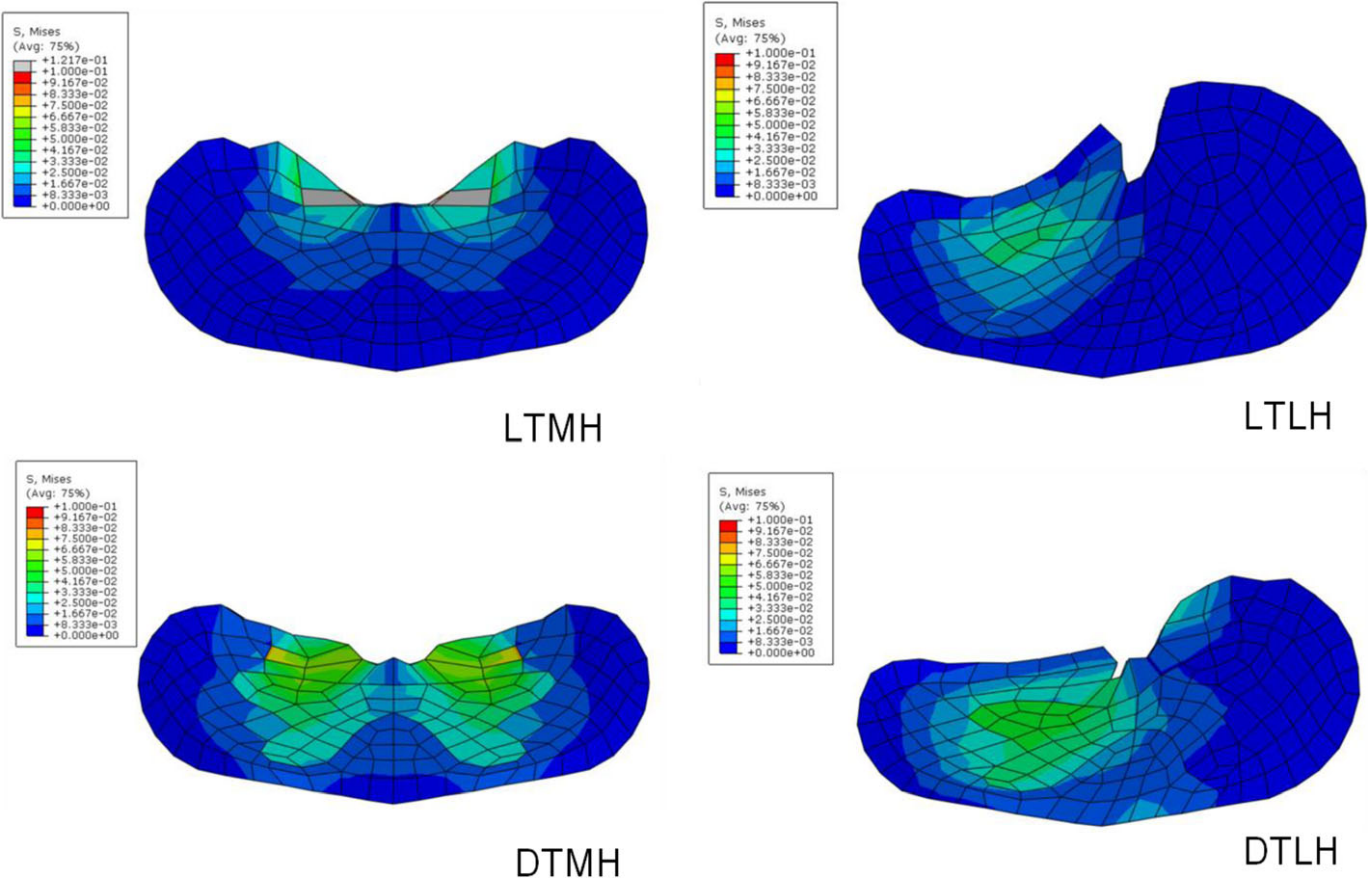
The distribution of von Mises stress in the middle transverse cross section of the gray matter and white matter in neutral position. LTMH, local type with median herniation; LTLH, local type with lateral herniation; DTMH, diffuse type with median herniation; DTLH, diffuse type with lateral herniation. Unit of stress: MPa

**Fig. 7:**
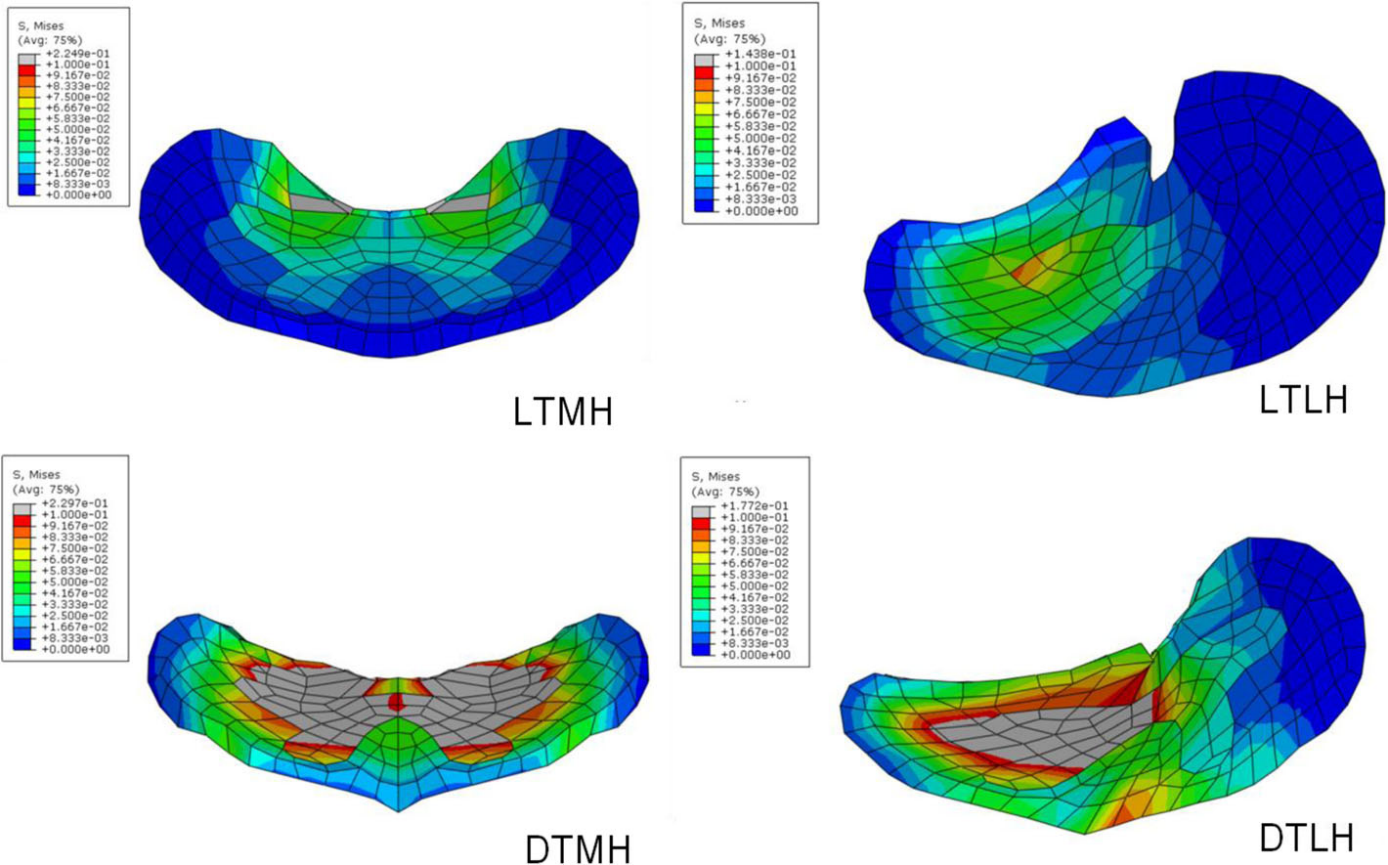
The distribution of von Mises stress in the middle transverse cross section of the gray matter and white matter in extension position. LTMH, local type with median herniation; LTLH, local type with lateral herniation; DTMH, diffuse type with median herniation; DTLH, diffuse type with lateral herniation. Unit of stress: MPa

The distribution of von Mises stress in the middle transverse cross section of the gray matter and white matter was presented in Fig. 6. and Fig. 7. In neutral position, the maximum von Mises stress was shown in white matter for local type, while it is in gray matter for diffuse type. Obviously, the higher von Mises stress and larger affected regions were shown in extension position than in neutral position for each load condition. Similar with neutral position, the gray matter was compressed seriously in extension position for diffuse type. And the von Mises stress maintained at a high level in the gray matter and a large area was influnced. The unified scale bar was used for each compression ratio for clear comparison.

## Discussion

A finite element model of cervical spinal cord was modeled. Local type with median herniation, local type with lateral herniation, diffuse type with median herniation, and diffuse type with lateral herniation were simulated in neutral and extention position. The maximum von Mises stresses in different components and the stress distributions in the middle transverse cross section were calculated four compression ratios.

Although preexisting severe cervical spinal cord compression is a significant risk factor for severe paralysis development in patients with traumatic cervical spinal cord injury without bone injury [13], it seemed that no liner correlation exists between the degree of preexisting compression and the severity of paralysis [13–15, 34–37]. Oichi et al. [13] investigated 122 patients with traumatic cervical spinal cord injury without bone injury. The degree of preexisting cervical spinal cord compression was divided into three categories in accordance with the maximum spinal cord compression (MSCC): minor compression (MSCC ≤ 20%), moderate compression (20% < MSCC ≤ 40%), and severe compression (40% < MSCC). Thus, the maximum displacement of the disc herniation model to the spinal cord was set to the 40% of the sagittal diameter of the spinal cord. In order to simulate acute protrusion of nucleus pulposus which is incompressible semi fluid due to hyperextension injury, the bulging disc was simulated as incompressible rigid body.

In this study, it was found that the maximum von Mises stresses to the ventral of spinal cord in extension position were much higher than in neutral position. In either the excessive flexion or extension positions, a tension will be produced on the convex side of the cervical spinal cord. This may aggravate the severity of spinal cord injury at the same degree of spinal cord compression [38], which is consistent with this study. Besides, the spinal canal diameter would be decreased with ligamentum flavum buckling in extension position, which could potentially aggravate compression, especially in patients with preexisting disc herniation. Delphine et al. [39] performed a retrospective case series study of 51 patients with spondylotic myelopathy. It was found that stages of stenosis (classification of Muhle et al. [40]) in the extension position were significantly higher than stages in the neutral and flexion positions. In the case of patients with partial obliteration of the subarachnoid space in the neutral position, it could show impingement of the spinal cord when patients extent to some degree.

In most cases, there was no impingement of the cord, and only in case of patients with partial obliteration of the subarachnoid space in the neutral position, it could show impingement of the cord in extension. Jha et al. [41] performed dynamic MRI on 66 patients with neck pain to determine the changes in cervical canal diameters and spinal cord compression. They found that the average spinal canal diameter showed significant decrease from flexion to extension position (*P* < 0.05) at each intervertebral level (from C2-3 to C7-T1). And higher stages in spinal cord compression were also found in extension position.

In Figs. 4, 5, 6 and 7, it was found that the local type of disc herniation mainly affected white matter while the diffuse type mainly affected gray matter. This result was consistent with Ono’s autopsy study [42]. In Ono’s study, the histologic finding of a 63-year-old males’ spinal cord, who suffered from severe cervical spondylotic myelopathy, showed marked flattening of the gray matter on both sides at the DTMH (C6 cord segment) and on the left side at the DTLH (C7 cord segment). Meanwhile, a remarkable neuronal loss or chromatolysis could be observed. However, the gray matter was relatively less affected than the white matter at the LTMH (C8 cord segment) and the LTLH (T1 cord segment). The average localized stress of the diffuse type was higher than that of the local type in our study. We speculate that this may be due to the larger space occupying effect and less reserve space of spinal canal in diffuse type than in local type. In addition, we found that the maximum von Mises stresses were mainly concentrated at the anterior horn and the peripheral white matter. In DTMH, the posterior horn was also involved. This explains the reason that acute central cord syndrome is the most commonly encountered type of the incomplete spinal cord injury in cervical hyperextension injury, characterized by a disproportionately more motor function in the upper extremities than in the lower ones, bladder dysfunction, and a variable amount of sensory loss below the level of injury. And DTMH is more prone to cause spinal cord injury under mild violence and with more severe neurological impairment.

The model has certain simplifications or assumptions. The preexisting severe cervical spinal cord compression includes disc herniation, osteophyma, and ligament calcification. In this study, we mainly focused on effect of the different types of disc herniation. Local type and diffused type were modeled by ball and pie geometry respectively. In actual situation, the geometries of the bulging discs are polymorphic. During the process of disc bulging, the geometry is changing. In this situation, a quantitative comparison analysis cannot be completed. Therefore, we assumed an ideal situation and keep the shape unchanged. Due to incompressibility of nucleus pulposus, the bulging discs were assumed as rigid. More detailed and complex bulging discs distinguished by normal and degenerative material properties may provide more interesting data in further study. The complete spinal canal, cerebrospinal fluid, and nerve roots were not modeled. Some parameter such as the change of spinal canal dimension cannot be evaluated. Future developments of the proposed FE model will include more anatomical structures to improve its effectiveness in simulating the stress field in cervical spinal cord regarding different pathological factors and positions.

## Conclusions

Cervical spinal cord with preexisting disc herniation is more likely to be compressed in hyperextension situation than in neutral position. Diffuse type with median herniation may cause more severe compression with higher von Mises stresses concentrated at the anterior horn and the peripheral white matter, resulting in acute central cord syndrome. More care should be taken to prevent cervical spine injury for the patients with preexisting severe disc herniation, especially in extension case.

## Data Availability

All data generated or analyzed during this study are included in the article.

## Abbreviations

FE: Finite element
LTMH: Local type with median herniation
LTLH: Local type with lateral herniation
DTMH: Diffuse type with median herniation
DTLH: Diffuse type with lateral herniation
MSCC: Maximum spinal cord compression
